# EMSA: Explainable multilingual sentiment analysis models providing sentiment analysis across multiple languages

**DOI:** 10.1371/journal.pone.0333508

**Published:** 2025-11-12

**Authors:** Li Zhao, Jinwei Zhou, Jinde Cao, Weina Zhu

**Affiliations:** 1 School of Information Science and Engineering, Yunnan University, Kunming, China; 2 Economics and Management School, Wuhan University, Wuhan, China; 3 School of Mathematics, Southeast University, Nanjing, China; Philadelphia University, JORDAN

## Abstract

Sentiment analysis across multiple languages remains a challenging problem due to linguistic diversity, domain-specific expressions, and the limited explainability of existing models. This study aims to address these issues by proposing the Explainable Multilingual Sentiment Analyzer (EMSA), a novel framework that integrates large language models with prompt engineering. EMSA employs a two-stage process, first generating sentiment reasoning through chain-of-thought prompts, and then producing sentiment classification with explicit interpretability. We evaluate EMSA on both the GubaSenti dataset (Chinese financial domain) and the SST dataset (English benchmark). Experimental results demonstrate that EMSA consistently outperforms pre-trained language models such as RoBERTa, XLNet, and ALBERT, while providing transparent reasoning steps that enhance user trust. These findings suggest that EMSA not only improves multilingual sentiment classification performance but also contributes to the development of more interpretable and practical sentiment analysis systems.

## 1 Introduction

In the last decade, artificial intelligence based on deep learning methods has made outstanding contributions to the progress of human society. Artificial intelligence can help humans make appropriate decisions in reasonable application scenarios [1]. With the rapid advancements in large language models (LLMs) in recent years, natural language processing has entered a new era. Following the introduction of the Transformer [2], a series of transformer-based pre-trained language models (PLMs) [3–9] have laid a strong foundation for natural language processing (NLP) tasks. By increasing both model size and training data, language models have effectively improved their performance and sample efficiency [10,11].

However, simply scaling up the model is insufficient to ensure optimal performance in more complex reasoning tasks such as arithmetic, commonsense, and symbolic reasoning [12]. To better harness the capabilities of language models for addressing these challenges, two commonly employed paradigms are training a blank deep network from scratch [13] or loading weights from a pre-trained model and fine-tuning it with downstream tasks [14]. The pre-training and fine-tuning paradigm has demonstrated its prowess in reasoning and gradually emerged as the dominant approach for NLP tasks. Prompt learning, a language model training strategy that involves guiding the model’s predictions by framing tasks as prompts, has also emerged based on this paradigm. Instead of fine-tuning the model with thousands of data samples, prompt learning only provides the model with a few input-output exemplars for demonstration purposes. While it can achieve performance close to that of the pre-training and fine-tuning paradigm, it fails to surpass it [15,16] primarily due to the data-driven nature of deep learning (i.e., lack of training samples poses a significant challenge). Despite falling short of surpassing the pre-training and fine-tuning paradigm, prompt learning still exhibits strong potential.

The advent of large language models in human-computer interaction has brought about a transformative shift. Due to the unprecedented scale of the model and the vast training data used, large language models such as the GPT series [11,17,18], LLaMA [19], and PaLM [20] possess strong reasoning abilities that were not present in previous pre-trained models. With the support of the LLMs, Chain-of-Thought prompting [21] draws inspiration from the step-by-step problem-solving approach of human thinking. It constructs cases by decomposing the problem into steps and achieves state-of-the-art performance across multiple inference tasks.

Sentiment analysis, a type of text classification task, requires models to examine the input text and determine the expressed sentiment. Specifically, models should identify whether the sentiment is positive, negative, or neutral, based on the text’s content. Sentiment analysis is currently a popular research direction in NLP domain, research towards sentiment analysis has recently been extensive [22–24], and it is widely applied in various aspects of social life [25]. Despite being considered one of the simplest natural language processing tasks, sentiment analysis does not hold a prominent position in current language model research. Pre-trained language models (PLMs) have demonstrated success in sentiment analysis tasks and can achieve accuracy rates exceeding 95% on public datasets like Standford Sentiment Treebank (SST) [26] binary sentiment analysis (SST-2).

However, there are still significant challenges to overcome. The challenge of sentiment analysis lies in the linguistic and cultural variations across languages, which strongly affect how sentiment is expressed and understood. Furthermore, sentiment datasets in many languages are scarce, which restricts the applicability of existing methods. Another important limitation is the lack of interpretability, most current models function as black boxes, providing predictions without explaining the reasoning process. These issues highlight a practical gap in current research, although existing models achieve strong accuracy in English, they fail to provide trustworthy and transparent solutions across diverse languages.

The motivation for this study arises from the need to address these gaps. We aim to design a framework that not only performs sentiment analysis effectively across multiple languages but also enhances interpretability. By combining the reasoning capabilities of LLMs with prompt engineering, our proposed Explainable Multilingual Sentiment Analyzer (EMSA) seeks to deliver accurate, multilingual sentiment classification along with transparent reasoning steps. This dual focus on multilingual robustness and explainability ensures greater trustworthiness and real-world applicability.

Our work makes the following key contributions:

1. Introduces a three-stage sentiment analysis framework: contextual interpretation, domain-specific adjustment, and reasoned classification, which transforms sentiment analysis from direct classification to structured reasoning, and enables traceable decision paths from input to final classification.
2. Develops a systematic methodology for incorporating domain-specific factors for stock market sentiment classification, which demonstrates effectiveness through integration of stock market trends in financial sentiment analysis.
3. Achieves state-of-the-art performance on both Chinese (GubaSenti) and English (SST) datasets, demonstrates language-independent reasoning architecture, and maintains consistent performance across linguistic boundaries.
4. Introduces GubaSenti, a new dataset containing 7,503,278 comments from the SSE Composite Index forum, includes 10,000 carefully annotated samples, which provides a challenging benchmark for evaluating multilingual sentiment analysis in the financial domain.

Related works are presented in Sect 2, whilst Sect 3 showcases the construction process of our GubaSenti dataset, Sect 4 presents a preliminary study demonstrating the importance of prompt engineering in sentiment analysis, and Sect 5 is devoted to the EMSA approach, while Sect 6 consists of results and analysis, then Sect 7 presents more detailed findings and further discussion, and Sect 8 concludes the paper.

## 2 Related works

This section reviews relevant studies on large language models, prompt engineering, and sentiment analysis evolution. Beyond summarizing prior approaches, we provide a critical analysis of their limitations and research gaps, highlighting why current methods are insufficient for multilingual and explainable sentiment analysis.

### 2.1 Large language models

After the widespread application of the PLMs, there has been significant progress in the NLP domain, leading to improved performance of language models on complex problems [27–29]. The LLMs, derived from PLMs, possess a powerful generation capability that early, smaller-scale PLMs lacked [30]. Unlike the pre-train, finetune and inference procedure for PLMs, the approach of LLMs for NLP tasks is through language model interface (e.g., ChatGPT interface), which eliminates the difference between research and engineering.

A previous study [31] shows that when LLMs reach a sufficient scale, they exhibit powerful reasoning and generation capabilities that were previously absent, which was defined as LLM’s emergent abilities. An indication of this capability arises when the performance of the model experiences a significant improvement upon reaching a certain scale. This phenomenon distinguishes LLMs from previous language models on complex language tasks such as language modeling and task solving, which require reasoning capabilities.

Although LLMs demonstrate strong general-purpose performance due to their large model capacity, their direct use for sentiment analysis without tailored prompt design remains inefficient and lacks interpretability. This limitation highlights the need for approaches that combine the reasoning abilities of LLMs with systematic prompt engineering to achieve both effective and explainable sentiment analysis.

**Fig 1:**
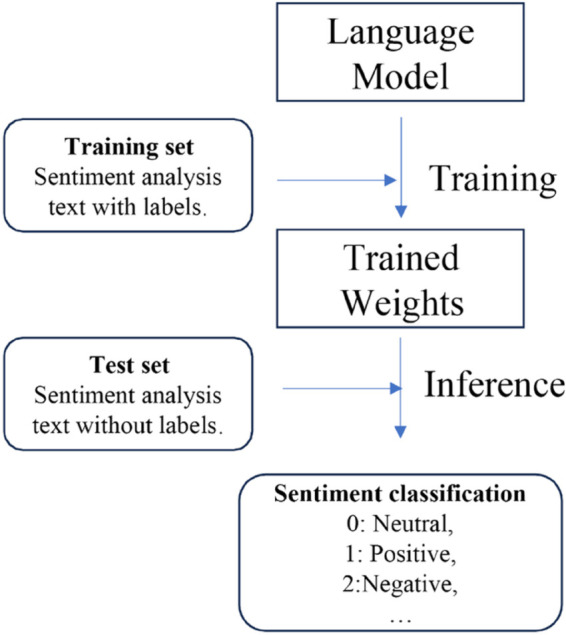
Training from scratch.

**Fig 2:**
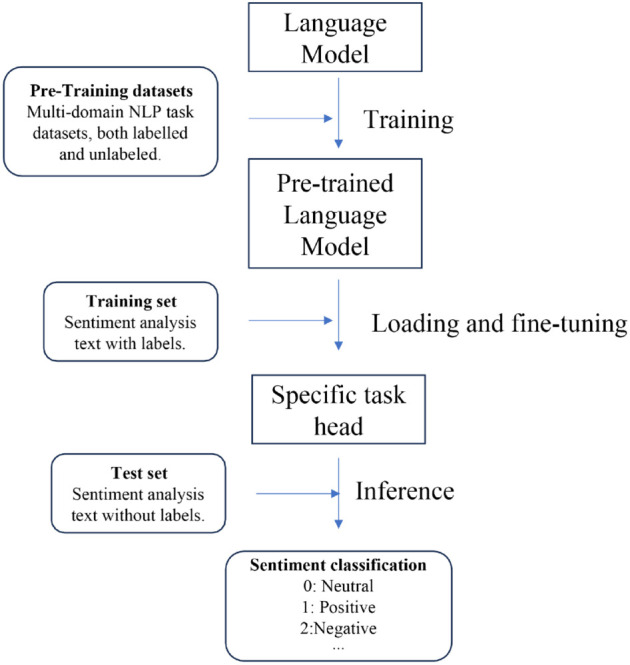
Pre-training and fine-tuning.

**Fig 3:**
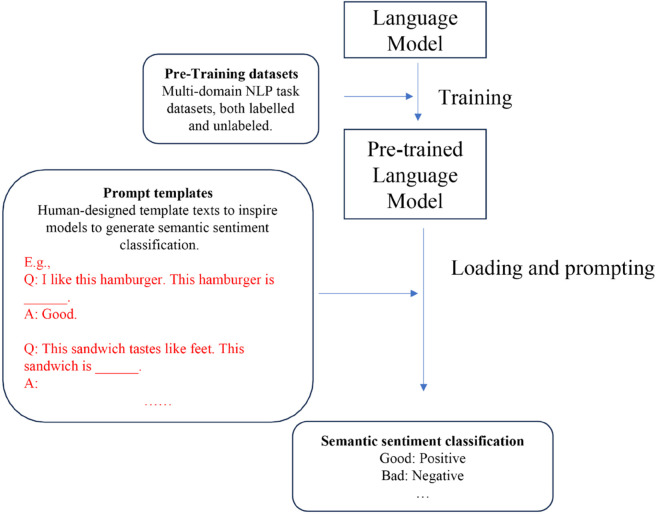
Prompt learning.

**Fig 4:**
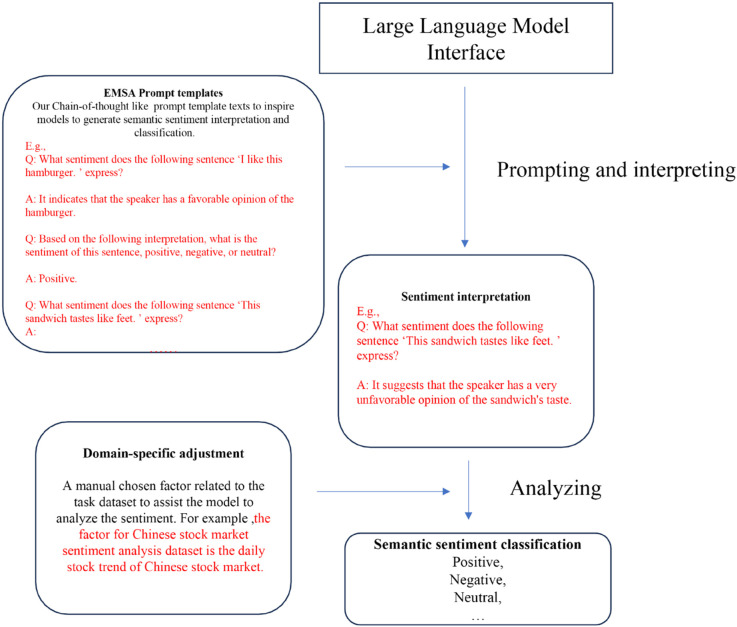
EMSA prompt engineering.

EMSA, as a prompt engineering approach, is developed based on LLMs. A series of LLMs such as GPT series [11], LLaMA [19], and PaLM [20] are applied to test the performance of our method. Unlike previous language models for sentiment analysis or text classification, EMSA employs a novel approach by dividing the sentiment analysis task into two distinct processes: sentiment reasoning and categorization. This division leverages the model’s reasoning capabilities more effectively, as it first deduces the underlying sentiment through reasoning, and then categorizes it accordingly. This method not only demands but also harnesses the advanced reasoning abilities of large language models, leading to a significant improvement in performance. By explicitly separating reasoning from categorization, EMSA taps into the inherent strengths of the models, thereby optimizing their analytical potential for more accurate sentiment analysis. As the models’ scale expands, their reasoning power increases, leading to enhanced performance of EMSA on the models, which is consistent with both the emergent abilities of LLM and our initial intuition. Figs 1–4 presents four NLP paradigms mentioned in this section.

### 2.2 Prompt engineering

Supervised NLP algorithms require extensive annotated datasets for training to achieve satisfactory task performance [27]. However, However, most real-world corpora consist predominantly of unlabeled data. Annotating significant volumes of data not only incurs prohibitively high costs but also complicates the evaluation of algorithms for tasks like text completion, where generating fresh content means there is no single correct label. The paradigm of annotating large volumes of data and then training models to solve the problem is not applicable to these tasks.

Due to extensive text input and multitask pre-training, PLMs demonstrate superior generalization capabilities compared to NLP models developed from scratch using deep learning techniques. Additionally, they can be fine-tuned for various downstream tasks to achieve superior performance. Recent advancements in the reasoning and generalization abilities of PLMs have led to the emergence of prompt engineering, a contemporary paradigm that eliminates the need for fine-tuning processes. Prompt engineering involves providing tailored prompts consisting of questions and answers to a model after loading pre-trained weights. For instance, in sentiment analysis tasks, an instance input *x*_1_ =  ’I love this sandwich.’ with answer *y*_1_ = ’It was a good sandwich.’ forms a complete prompt instance $\left(x_{1},y_{1}\right)$ = ’I love this sandwich. It was a good sandwich.’ The given question *x* = ’I dislike this hamburger.’ and answer template *y* = ’It was a hamburger.’ prompt engineering transforms the sentiment analysis task from predicting binary labels (e.g., 0 for negative and 1 for positive) to analyzing the sentiment conveyed by the input sentence and generating a semantically appropriate response. These algorithms based on prompt engineering can achieve performance comparable to that of pre-training and fine-tuning approaches, but they fall short of reaching the state-of-the-art level [15,32,33].

The extensive utilization of LLMs provides an opportunity for a transformative shift in prompt engineering-based methods. Chain- of-Though (CoT) [21], a prompt engineering approach that instructs LLMs to emulate the human reasoning process by thinking step by step, has achieved state-of-the-art performance in tasks such as arithmetic reasoning [14,34–36] and common-sense reasoning [37–40]. Building upon CoT, a series of prompt engineering approaches have surpassed pre-training and fine-tuning methods in various NLP tasks [41–45], signifying a new era for LLMs where they possess the potential to reason like humans and solve complex problems.

Despite the remarkable progress of prompt engineering in areas such as mathematical reasoning, logical inference, and machine translation, its application to sentiment analysis has been relatively limited. Current research seldom explores how advanced prompt engineering strategies could enhance both the performance and interpretability of sentiment analysis tasks, particularly in multilingual contexts.

Our approach fully leverages the reasoning capability of LLMs by transforming the sentiment analysis task from generating sentiment categorization outputs to engaging in the process of analyzing sentiment through reasoning and subsequently producing classifications. With our EMSA, we achieve both satisfactory classification performance and provide robust explanations to the final outputs, thereby enhancing the persuasiveness and explainability of sentiment analysis to some extent.

### 2.3 Evolution of sentiment analysis approaches

The development of sentiment analysis has undergone several paradigm shifts, from pre-trained language models (PLMs) to the current era of large language models (LLMs) with prompt engineering. Each stage has brought distinct advantages and methodological innovations to the field.

The introduction of PLMs marked a significant advancement in sentiment analysis. BERT [3] and its variants demonstrated that pre-training on large-scale corpora followed by task-specific fine-tuning could significantly improve sentiment classification performance. RoBERTa [7] further enhanced this approach through optimized pre-training strategies, while XLNet [9] introduced permutation language modeling to capture bidirectional context more effectively. These models achieved impressive results on standard benchmarks, with ALBERT [4] reaching 95.9% accuracy on SST-2 [26] through parameter-efficient architecture design.

A crucial development in this era was the emergence of sentiment-specific pre-trained models. SKEP [46] introduced sentiment knowledge enhanced pre-training, explicitly incorporating sentiment information during the pre-training phase. By integrating sentiment-specific features and employing specially designed pre-training tasks, SKEP achieved state-of-the-art performance across various sentiment analysis benchmarks, reaching 96.7% accuracy on SST-2. Similarly, SentiLARE [47] advanced the field by incorporating linguistic knowledge and sentiment lexicons into the pre-training process. This sentiment-aware language representation approach demonstrated superior performance in capturing subtle sentiment expressions and dealing with complex sentiment contexts, achieving 96.5% accuracy on SST-2. These specialized models showed that incorporating sentiment-specific knowledge during pre-training could provide significant advantages over general-purpose language models.

The next evolutionary stage saw the emergence of prompt-tuning approaches, which began bridging the gap between pre-training and task-specific requirements. Rather than modifying the entire model through fine-tuning, prompt-tuning methods like P-tuning [48] and Prefix-tuning introduced learnable prompt embeddings. These approaches reduced the parameter count for adaptation while maintaining competitive performance. The prefix-tuning approach demonstrated particular promise in sentiment analysis by learning task-specific prefixes that could effectively guide the model’s predictions.

The advent of large language models marked another transformative shift. Models like GPT-3 [11] demonstrated remarkable few-shot learning capabilities through prompt engineering, eliminating the need for task-specific training or tuning. This capability was further enhanced by PaLM [20] and GPT-4, which showed increasingly sophisticated understanding of sentiment across different domains and languages. The key innovation lay in their ability to perform complex reasoning through carefully designed prompts, rather than requiring task-specific architectural modifications.

This evolutionary trajectory highlights important research gaps in sentiment analysis. Although traditional fine-tuning and sentiment-specific pre-training (e.g., SKEP, SentiLARE) have achieved strong results, recent advances in prompt engineering and large language models have given relatively little attention to sentiment analysis. Most studies still focus on English benchmarks, overlooking the challenges in non-English languages and domain-specific contexts. Consequently, there remains a significant gap in developing explainable and effective sentiment analysis methods that extend beyond English and address diverse multilingual and domain-specific scenarios.

### 2.4 Explainable Multilingual Sentiment Analyzer (EMSA)

Existing research demonstrates significant progress in NLP, yet several gaps remain. Large language models show strong reasoning abilities but lack efficiency and interpretability without careful prompt design. Prompt engineering has achieved breakthroughs in reasoning tasks but is underexplored in sentiment analysis, particularly across languages. Furthermore, while sentiment-specific pre-training has achieved high performance on English benchmarks, it often neglects multilingual and domain-specific contexts. These gaps provide the foundation and motivation for our proposed EMSA framework, which aims to combine multilingual robustness with transparent and explainable sentiment reasoning.

EMSA operates on the LLM interface and utilizes CoT [21]-like prompt templates to guide the LLM interfaces in solving sentiment analysis tasks step by step. It first generates a sentiment interpretation and then outputs a sentiment classification based on this interpretation. This procedure not only achieves superior sentiment classification performance but also enhances model explainability by allowing users to trace the entire analysis process. Additionally, EMSA incorporates domain-specific adjustments (e.g., for GubaSenti, a Chinese stock market sentiment analysis dataset) based on the daily trend of the Chinese stock market to assist LLMs in generating more convincing interpretations and improving classification performance.

In contrast to prior studies, this work makes a unique contribution by proposing an explainable multilingual sentiment analysis (EMSA) framework. Unlike existing methods that focus mainly on improving classification accuracy, the EMSA framework emphasizes both performance and interpretability across multiple languages. By providing transparent explanations for model predictions, our approach not only ensures robust multilingual performance but also enhances user trust and applicability in real-world scenarios. This dual focus on multilingual generalization and explainability distinguishes our study from previous research in the field.

## 3 Datasets

### 3.1 GubaSenti dataset

The data-driven nature of deep learning models dictates that large-scale datasets are always the optimal choice for model training and data analysis. However, our research has revealed that text data extracted from Chinese stock market forums possesses valid properties suitable for sentiment analysis. Firstly, the sheer volume of Chinese text available in these forums is substantial enough to construct a sufficiently large sentiment analysis dataset. Secondly, the textual content found within stock market forums encompasses a wide range of emotions, thereby increasing the complexity of sentiment analysis and providing an ideal platform for evaluating algorithm performance. Lastly, it is worth noting that existing LLMs predominantly rely on English datasets during pre-training phases. Consequently, utilizing Chinese datasets presents a more challenging scenario for models and enables evaluation of their capability to perform sentiment analysis in multilingual environments.

The GubaSenti dataset is constructed from the SSE Composite Index forum on East Money, one of China’s largest financial information platforms. We collected a total of 7,503,278 comments spanning from January 2011 to October 2023, ensuring broad coverage of market cycles and investor sentiments.

The journey from raw data to a high-quality sentiment analysis dataset involved several careful preprocessing steps. Beginning with our initial collection of 7,503,278 comments, we first eliminated duplicate entries to ensure data independence. We then applied length constraints, removing extremely short comments (fewer than 3 Chinese characters) that typically lack meaningful sentiment and very long comments (exceeding 100 characters) that often contain irrelevant information. Further refinement involved removing non-textual elements such as images and URLs, as well as filtering out automated bot messages and promotional content. This thorough preprocessing resulted in a refined pool of 5,826,453 valid comments, each containing meaningful, human-generated market commentary.

The annotation process demanded particular attention to ensure high-quality sentiment labels. We assembled a team of ten highly qualified annotators, each bringing substantial financial expertise to the task. Our annotators were selected based on three key criteria: native Chinese language fluency combined with formal financial education (minimum of a bachelor’s degree in finance or economics), significant practical experience (at least two years) in financial markets, and demonstrated competence in sentiment analysis through a preliminary test where they needed to achieve over 90% agreement with expert annotations.

To create a manageable yet representative dataset, we carefully selected 10,000 comments from our preprocessed pool. This selection process wasn’t merely random; we employed a stratified sampling approach to maintain the temporal distribution patterns of the original dataset, ensure balanced representation of both bull and bear market conditions, and preserve the natural variety in comment lengths. This careful sampling strategy helps ensure our final dataset captures the full complexity of market sentiment expressions across different market conditions.

All the following experiments of our proposed prompt engineering methodology are conducted on the 10,000 annotated samples. Final dataset composition and characteristics are shown in Table 1. The resulting dataset provides a robust foundation for sentiment analysis research, with strong inter-annotator agreement and comprehensive documentation of the annotation process. Table 2 showcases 10 samples from the annotated dataset.

**Table 1:** GubaSenti dataset statistics.

Characteristic	Value
Total valid comments	10,000
Positive sentiment	3,438 (34.3%)
Negative sentiment	3,896 (39.0%)
Neutral sentiment	2,666 (26.7%)
High agreement (> 80%)	7,245 (72.4%)

**Table 2:**
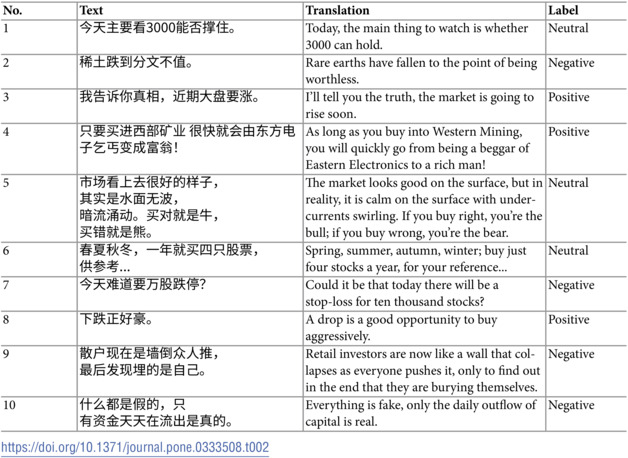
10 examples of GubaSenti dataset.

### 3.2 Stanford Sentiment Treebank (SST) dataset

In addition to GubaSenti, which focuses on Chinese stock market discussions, we also employ the Stanford Sentiment Treebank (SST) [26], one of the most influential benchmark datasets in English sentiment analysis. SST is constructed from movie reviews and provides sentiment annotations at both the phrase and sentence levels. A unique feature of SST is that it is built upon a fully parsed syntactic tree, allowing each phrase in a sentence to carry a sentiment label. This fine-grained annotation scheme enables evaluation across multiple levels of granularity.

The dataset is available in two main settings: a binary classification task (positive vs. negative) and a fine-grained five-class classification task (very negative, negative, neutral, positive, very positive). This flexibility has made SST a cornerstone in sentiment analysis research, as it supports comprehensive evaluation of models’ ability to capture subtle sentiment distinctions. Due to its widespread adoption, SST serves as a common benchmark for comparing sentiment classification models in English.

By including both GubaSenti and SST, we ensure that our evaluation covers two complementary perspectives: domain-specific Chinese sentiment in financial contexts, and general-purpose English sentiment in movie reviews. This combination highlights the multilingual and cross-domain applicability of our EMSA framework, providing a more rigorous and balanced evaluation.

## 4 Preliminary study

### 4.1 Evaluation metrics

We evaluate the performance of the proposed framework using widely adopted metrics in classification tasks, including accuracy, precision, recall, and the F1-score. These metrics provide a comprehensive view of the model’s ability to correctly classify instances, balance false positives and false negatives, and achieve overall predictive reliability.

### 4.2 Dataset

The GubaSenti dataset is utilized for algorithm evaluation. The 10,000 annotated data points are divided into a training set and a test set in a ratio of 9:1. The training set is employed to fine-tune the pre-trained models, while the fine-tuned models are ultimately evaluated on the test set. To ensure more stable results, we employ a 10-fold validation approach. As for the prompt engineering approach, which does not involve any training or fine-tuning process, it is directly assessed on the test set for comparison purposes.

### 4.3 Prompt engineering Vs. Pre-training and fine-tuning

#### 4.3.1 Pre-training and fine-tuning models.

In this preliminary study, we select BERT [3], RoBERTa [7], XLNet [9], and ALBERT [4] as baseline models, since they represent four widely-adopted architectures in the family of Transformer-based pretrained models. BERT introduces bidirectional encoding via masked language modeling, offering strong contextual representations. RoBERTa improves upon BERT by leveraging larger-scale data and dynamic masking, which generally enhances robustness in sentiment classification. XLNet adopts a permutation-based training objective that better captures long-range dependencies, which is particularly beneficial for nuanced sentiment expressions. ALBERT, by employing parameter sharing and factorized embeddings, achieves efficiency while retaining competitive performance, making it suitable for comparative analysis. By including these models, we aim to cover a broad spectrum of architectural innovations, enabling a meaningful comparison with our prompt-based methods.

The four models are loaded and fine-tuned. Each model was loaded with multiple weight sizes, including the base model and large model, and then fine-tuned using the Adam optimizer [49]. The training was conducted for a maximum of 10 epochs, with a maximum sequence length of 50, a batch size of 32, and a learning rate of 0.1, 0.01, 0.001, or 0.0001 for grid search purposes (0.001 tended to yield the best performance).

#### 4.3.2 Prompt engineering approach.

**Fig 5:**
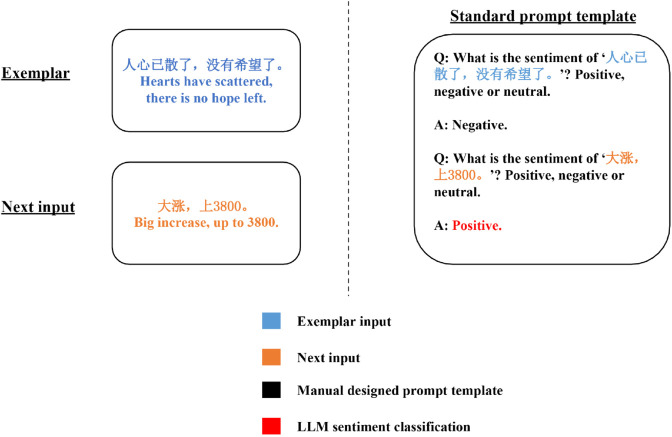
An example of 1-shot standard prompt template.

We use a 1-shot standard prompt template containing a single example for prompt engineering. As shown in Fig 5, an exemplar is a 1-hop process consisting of a question and an answer, the question asks what is the sentiment of the input text, and the answer only contains the sentiment class (positive, neutral, or negative). The LLM used for prompt engineering is GPT-3.5-turbo [11].

As shown in Table 3, the standard prompt exhibits superior performance on the GubaSenti dataset, outperforming RoBERTa-base, which is considered the best-performing model among pre-training and fine-tuning models in terms of accuracy (+14.6%), precision (+15.3%), recall (+17.8%), and F1-score (+14.5%). These results demonstrate the potential of prompt engineering to achieve high performance in sentiment analysis at a low cost, thereby validating and justifying our study. Mode detailed quantitative comparison between our prompt engineering approach and the former PLM SOTA methods is presented in Sect 5.

**Table 3:** Comparison of pre-training and fine-tuning models and prompt engineering on GubaSenti sentiment analysis on the accuracy, precision, recall and F1-score (%).

Method	Accuracy	Precision	Recall	F1
BERT-base [3]	43.6	43.2	43.2	41.8
RoBERTa-base [7]	50.0	48.8	50.5	48.2
XLNet-base [9]	49.4	49.7	50.5	46.3
ALBERT-base [4]	40.9	40.6	41.4	37.8
Standard prompt	**64.6**	**64.1**	**68.3**	**62.7**

### 4.4 Prompt template selection

**Fig 6:**
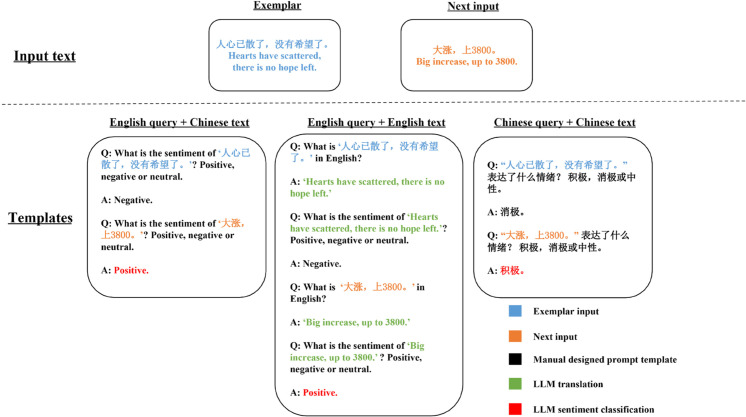
Prompt templates with varied combinations of English and Chinese.

Considering that English serves as the predominant language employed in developing and training AI models, especially those about to NLP domains, a substantial volume of research data exclusively exists in English. As a result, numerous prompt engineering approaches rely on English templates and text. These circumstances give rise to various inquiries regarding our Chinese-based GubaSenti dataset: How can we devise an optimal prompt template that attains peak performance? Is it imperative to translate Chinese text into English for LLMs? Which language should be utilized when querying the LLM interface? To tackle these concerns comprehensively, we have formulated three distinct exemplary prompt templates aimed at comparing their performances. These templates consist of different combinations of English and Chinese with query and input text (see Fig 6). The pure English template (English manual designed prompt template+ English exemplar input) performs a process contains a translation task from Chinese to English, and the translated text is used for the sentiment classification task. Both tasks are accomplished by our chosen LLM. Experiments on this phase are conducted on GPT-3.5-turbo.

As demonstrated in Table 4, employing a template with Chinese query and Chinese text yields the highest scores across all four metrics, indicating the benefits of designing prompt templates in the same language as the target dataset. Consequently, for our subsequent experiments on GubaSenti dataset, we exclusively employ prompt templates designed in Chinese, while for other datasets, we design prompt templates based on their original languages.

**Table 4:** Comparison between 3 prompt templates on GubaSenti sentiment analysis on the accuracy, precision, recall, and F1-score (%).

Method	Accuracy	Precision	Recall	F1
En_Template + Cn_Input	64.6	62.7	64.1	67.9
En_Template + En_Input	63.6	63.8	64.0	66.4
Cn_Template + Cn_Input	**65.4**	**64.4**	**66.0**	**68.3**

Note: En_Template represents an English manual designed prompt template, while Cn_Template is a Chinese manual designed prompt template. En_Input is the English exemplar input, and Cn_Input is the Chinese exemplar input.

## 5 EMSA prompt method

**Fig 7:**
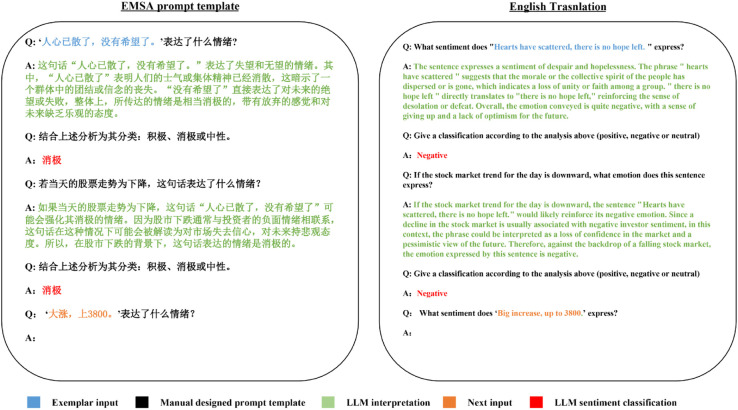
EMSA propmt example. All of the content used to construct the template is generated by GPT-4 API and the correctness and effectiveness of the generated content have been verified manually. (The English translation is only an English version of the EMSA prompt template and it is not relevant to the experiment.)

The task of sentiment analysis, widely regarded as the most straightforward NLP task, has traditionally employed simplistic prompt engineering approaches. These approaches typically involve designing 1-hop question templates [15,32], consisting solely of a question (input text) and an answer (sentiment class), as depicted in Fig 5. In contrast, our proposed EMSA approach treats sentiment analysis as a comprehensive process encompassing detailed interpretation of input text, adjustment based on domain-specific factors, and sentiment classification. Through our EMSA prompt engineering technique, we not only enhance the performance of sentiment classification but also ensure semantic explainability and facilitate error analysis and troubleshooting. An illustrative example of EMSA is presented in Fig 7.

### 5.1 Chain-of-thought like prompting

By decomposing input questions into sequential steps and solving them iteratively, CoT [21] has achieved remarkable success across various NLP tasks; however, its application in sentiment analysis remains unexplored. This is primarily attributed to the fact that a standard sentiment analysis dataset typically comprises only an input text and a classification label, lacking the feasibility of step-by-step resolution. To address this limitation, we propose a novel approach for sentiment analysis by reevaluating the analytical process. We break down the reasoning procedure into four distinct stages: comprehensive interpretation of sentiments within the input text, sentiment classification based on this interpretation, adjustment of interpretation considering domain-specific factors we have designed, and ultimately classification based on the refined interpretation. These three steps collectively form a coherent chain of reasoning that enables seamless integration of the CoT prompt template into sentiment analysis tasks.

### 5.2 Sentiment interpretation

The sentiment analysis tasks involve converting target texts into word vectors and feeding them into deep networks through either training from scratch or pre-training fine-tuning paradigms. The classification results are obtained by adjusting the weights of each node in the network using the task head, thereby enhancing the overall classification performance. However, it is worth noting that apart from the final classification result, most of the intermediate processes within the neural network are represented by vectors of varying sizes, which lack traceability and interpretability. In cases where algorithms perform poorly, researchers often resort to continuously tuning hyperparameters to identify an optimal combination. The process is highly time-consuming, and when dealing with different datasets, the hyperparameters require readjustment. The exceptional reasoning ability of LLMs enables an enhancement in the interpretability of sentiment analysis tasks. EMSA requests detailed interpretations of sentiment from the LLM interface based on text inputs. In case of errors in sentiment classification, it becomes possible to trace back to the comprehensive interpretation of the input statement to assess its reasonableness. This approach ensures explainability and feasibility in fine-tuning prompt exemplars.

### 5.3 Domain-specific adjustment

Our proposed GubaSenti is a Chinese stock sentiment analysis dataset. Unlike most open-source datasets widely utilized in deep learning algorithms, the pre-training fine-tuning paradigm exhibits suboptimal performance on the GubaSenti dataset as demonstrated in Sect 3. The linguistic expressions found in stock forums significantly deviate from everyday language, and over the past few years, sarcasm has become extensively prevalent across various forums. The high error rate persists even when human judgment is employed for sentiment analysis of these statements. We contend that the stock market trends effectively contribute to sentiment analysis. Depending on the fluctuations in the stock market, a single sentence can possess diametrically opposite connotations. Consequently, we have compiled the daily overall trends (rise or fall) of the Chinese stock market from January 2011 to October 2023 and incorporated them into sentiment interpretation. Subsequently, LLMs are requested to reassess the sentiment of input text based on the daily trend of the stock market and adjust their final sentiment classification accordingly.

### 5.4 Novel contributions and differentiation from existing methods

As shown in Table 5, While EMSA builds upon the foundation of chain-of-thought (CoT) prompting, it introduces several key innovations specifically designed for sentiment analysis tasks. The evolution from standard prompting to EMSA represents a significant advancement in reasoning complexity and effectiveness. Standard prompting employs a simplistic single-step classification approach, where the model directly maps input text to sentiment labels without intermediate reasoning. Chain-of-thought introduces basic logical deduction, allowing for simple explanations of the classification process. EMSA, however, implements a sophisticated multi-stage analysis framework that fundamentally transforms the sentiment analysis task.

**Table 5:** Comparison of different prompting approaches.

	Standard Prompting	Chain-of-Thought	EMSA
**Example**	Q: Sentiment? A: Positive	Q: Sentiment? Step 1: Contains positive word A: Positive	Q: Sentiment? Step 1: Interpret context Step 2: Consider domain factors Step 3: Classify with reasoning
**Reasoning**	Single-step classification	Basic logical deduction	Multi-stage analysis
**Context**	Text only	Text and basic context	Full domain integration
**Verification**	End result only	Single reasoning step	Multiple checkpoints
**Adaptability**	Fixed structure	Limited adaptation	Domain-specific flexibility

EMSA’s reasoning structure encompasses three key stages. First, the contextual interpretation stage performs deep analysis of the text’s sentiment implications. Second, the domain factor integration stage systematically incorporates domain-specific context. Finally, the reasoned classification stage produces the final classification based on comprehensive analysis of the previous stages.

A crucial differentiation lies in how each method handles contextual information. Standard prompting operates solely on the input text, disregarding any external context. Chain-of-thought advances this by considering basic contextual clues within the text. EMSA takes a significant step forward by systematically integrating domain-specific factors, enabling more nuanced and accurate analysis.

For instance, in financial sentiment analysis, EMSA can incorporate market trends, company performance metrics, and broader economic indicators into its reasoning process. This capability is particularly valuable in domains where sentiment is heavily influenced by external factors.

The approaches differ significantly in their ability to verify and validate sentiment classifications. Standard prompting provides only the final classification, offering no means for verification. Chain-of-thought presents a single reasoning step that can be verified. EMSA introduces multiple verification checkpoints throughout its analysis pipeline. This multi-checkpoint system enables comprehensive quality control at various stages. It allows for early detection of reasoning errors and validation of contextual interpretations. The system also facilitates verification of domain factor integration and confirmation of classification consistency. This layered approach to verification significantly enhances the reliability of the final sentiment analysis.

The adaptability of each method to different domains and contexts varies substantially. Standard prompting maintains a fixed structure regardless of domain, while chain-of-thought offers limited adaptation through general reasoning. EMSA distinguishes itself by providing systematic domain-specific flexibility.

The structural differences between these approaches have significant practical implications. In terms of accuracy, EMSA’s multi-stage analysis reduces error propagation, while its domain factor integration improves contextual understanding. The multiple verification points further enhance reliability of the results.

Regarding explainability, EMSA’s clear reasoning stages facilitate understanding of the classification process. The domain context integration provides strong justification for decisions, while the verification points offer transparency throughout the analysis pipeline. These innovations collectively enable EMSA to achieve superior performance while maintaining explainability and adaptability across different domains and languages.

### 5.5 Dataset compliance statement

All datasets used in this study are publicly available benchmark resources. The SST datasets are released by Stanford University under terms that permit academic research use. The GubaSenti dataset was compiled exclusively from publicly available online financial discussions, with collection and analysis fully compliant with the terms and conditions of the source platform. No private or personally identifiable information was collected or analyzed in this study.

The collection and analysis method complied with the terms and conditions for the source of the data.

## 6 Experiments and results

The effectiveness and capability of EMSA in multilingual sentiment analysis tasks are validated through experiments conducted on the GubaSenti dataset (Chinese) and the SST dataset (English). All experiments are performed using LLM APIs without any network training process. The prompt engeering experiments are conducted on an AMD Ryzen 5800 CPU, Python 3.8, and Openai 4.0.0. As no training process is involved, GPUs are not used. The fine-tuning process of former SOTA models are conducted on a single Nvidia GeForce RTX 3090 GPU with 24 GB RAM.

Multiple former state-of-the-art methdos, including general pre-trained models, sentiment-specific models, nad prompt-based methods are chosen as baselines, as shown in Table 6. We maintain the raw training configurations while fine-tuning the baseline models.

**Table 6:** Overview of baseline models for sentiment analysis comparison.

Category	Model	Architecture	Key Characteristics
General Pre-trained Models	RoBERTa-Large [7]	Transformer	Optimized training procedure, dynamic masking, 355M parameters
	XLNet-Large [9]	Transformer-XL	Permutation language modeling, broader context modeling
	ALBERT-xxlarge [4]	Transformer	Parameter-efficient architecture, cross-layer parameter sharing
Sentiment- Specific Models	SKEP [46]	BERT-based	Sentiment knowledge enhanced pre-training, explicit sentiment features
	SentiLARE [47]	RoBERTa-based	Incorporates word-level sentiment polarity, lexicon-based enhancement
Prompt- Based Methods	Standard Prompting	GPT-3.5	Direct sentiment classification through natural language prompting
	Chain-of-Thought [21]	GPT-3.5	Step-by-step reasoning through decomposed prompting strategy

### 6.1 Performance on GubaSenti dataset

**Fig 8:**
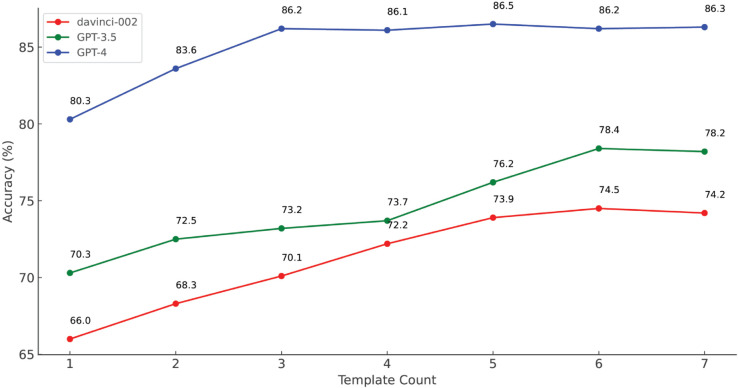
Accuracy of multiple GPT models on GubaSenti with different number of templates.

We conduct experiments to evaluate the most appropriate number of templates when applying the EMSA to the LLM interface. Since the question template of EMSA is designed to be multi-hop including sentiment classification and interpretation of a whole sentiment process, we consider zero-shot prompting as not applicable. As shown in Fig 8, both Davinci-002 and GPT-3.5 achieve their best performance with six cue templates, while the performance of GPT-4 peaks when the number of templates is three and shows no significant variation when the number exceeds three. Thus, we set the template number of EMSA to 6 as the default so that all 3 LLMs can have decent performance. As each text sample of GubaSenti has three classes (positive, neutral and negative), the default six templates consist of two positive, two neutral, and two negatives. In contrast to the experiments conducted on GubaSenti in Sect 3, we utilize all 10,000 annotated data samples from GubaSenti for evaluation purposes. Please refer to Sect 3.

Table 7 presents a comprehensive evaluation of our proposed EMSA method against various baseline models on the GubaSenti dataset. The results demonstrate significant performance improvements across all evaluation metrics, revealing several important patterns in model capabilities and the effectiveness of different approaches to sentiment analysis.

**Table 7:** Performance comparison on GubaSenti dataset.

Category	Model	Accuracy	Precision	Recall	F1
General Pre-trained	RoBERTa-Large	56.7	57.0	57.0	55.2
	XLNet-Large	54.0	53.5	52.6	51.8
	ALBERT-xxlarge	58.1	57.9	57.4	58.0
Sentiment- Specific	SKEP	61.5	60.9	61.2	61.1
	SentiLARE	62.3	61.8	62.0	61.9
Prompt- Based	Standard + text-davinci-002	61.8	62.3	62.1	63.9
	Standard + GPT-3.5	66.3	66.2	65.8	65.4
	Standard + GPT-4.0	79.6	80.5	78.8	78.7
	CoT + text-davinci-002	74.5	75.5	74.3	73.9
	CoT + GPT-3.5	78.4	78.2	78.0	79.6
	CoT + GPT-4.0	84.6	85.0	85.6	85.2
Ours	EMSA + text-davinci-002	74.5	75.5	74.3	73.9
	EMSA + GPT-3.5	78.4	78.2	78.0	79.6
	EMSA + GPT-4.0	**86.2**	**86.6**	**87.4**	**86.4**

The general pre-trained models show relatively limited performance on this challenging dataset. Among these, ALBERT-xxlarge achieves the best performance with an F1 score of 58.0%, followed by RoBERTa-Large at 55.2% and XLNet-Large at 51.8%. These results suggest that general pre-training alone, despite the sophisticated architecture of these models, is insufficient for handling the complexities of domain-specific Chinese financial sentiment analysis.

Moving to sentiment-specific models, we observe modest improvements in performance. SentiLARE and SKEP achieve F1 scores of 61.9% and 61.1% respectively, showing gains of approximately 3.9% over the best general pre-trained model (ALBERT-xxlarge). While this improvement indicates the value of sentiment-specific pre-training, these models still fall considerably short of prompt-based methods, highlighting the limitations of traditional approaches in handling complex financial sentiment expressions.

The prompt-based methods demonstrate a clear progression in performance across different LLM versions and prompting strategies. With standard prompting, we see a steady improvement from text-davinci-002 (63.9% F1) to GPT-3.5 (65.4% F1), with GPT-4.0 showing a substantial jump to 78.7% F1. This progression indicates the significant impact of model scale and architecture improvements on sentiment analysis capability.

Chain-of-Thought (CoT) prompting further enhances performance across all models. Text-davinci-002 with CoT achieves 73.9% F1, marking a significant 10.0% improvement over standard prompting. This improvement pattern continues with GPT-3.5 (79.6% F1, +14.2% over standard) and GPT-4.0 (85.2% F1, +6.5% over standard), demonstrating the effectiveness of structured reasoning in sentiment analysis tasks.

Our EMSA approach shows compelling results across different LLM versions. With text-davinci-002 and GPT-3.5, EMSA achieves performance comparable to CoT prompting, reaching F1 scores of 73.9% and 79.6% respectively. However, the combination of EMSA with GPT-4.0 yields the best overall performance, achieving an F1 score of 86.4%. This represents a significant improvement of 28.4% over the best general pre-trained model, 24.5% over the best sentiment-specific model, 7.7% over standard prompting with GPT-4.0, and 1.2% over CoT prompting with GPT-4.0.

Several key patterns emerge from these results. First, model scale consistently influences performance, with larger language models demonstrating superior capabilities in sentiment analysis. Second, advanced prompting strategies (both CoT and EMSA) significantly outperform standard prompting, particularly when combined with more capable models. Third, EMSA’s superior performance validates the effectiveness of our multi-stage approach in handling domain-specific sentiment analysis. Finally, the synergistic effect between EMSA and GPT-4.0 suggests that our method effectively leverages both architectural improvements and enhanced model capabilities.

The strong performance of EMSA across different metrics also demonstrates its robustness. The high precision (86.6%) and recall (87.4%) scores achieved with GPT-4.0 indicate balanced performance across different sentiment categories, while the consistent improvement pattern across model versions suggests stable and reliable enhancement over baseline approaches.

These comprehensive results validate EMSA’s design choices and demonstrate its effectiveness in complex, domain-specific sentiment analysis tasks. The significant improvements over strong baselines, particularly in combination with advanced language models, suggest that EMSA successfully addresses the challenges of financial sentiment analysis while providing a framework that can effectively utilize the capabilities of increasingly powerful language models.

**Fig 9:**
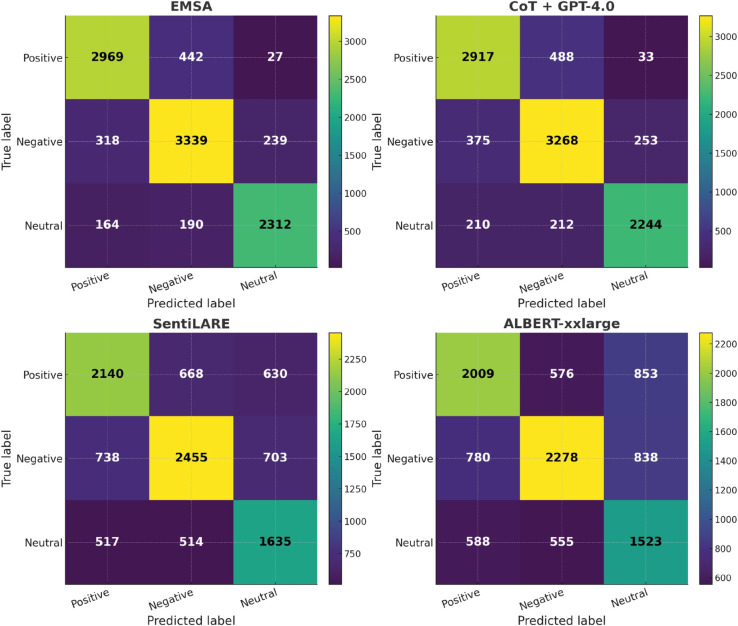
Confusion matrices for Gubasenti.

To provide a more intuitive view of classification tendencies, we additionally present confusion matrices for the best performed models of each category. On GubaSenti (Fig 9), all prompt engineering methods achieve strong performance, with only minor confusion between positive and negative classes, with our method achieves the best overall performance, which is consistent with the above observations.

### 6.2 Performance on SST dataset

To explore EMSA’s potential for multilingual-based sentiment analysis tasks, we conduct experiments on the SST dataset [26], an English-based sentiment analysis dataset. As is widely used as a benchmark for language models, the SST dataset normally has two tasks, namely a binary (positive or negative) sentiment classification task (SST-2) and a 5-class (positive, slightly positive, neutral, slightly negative, or negative) sentiment classification task (SST-5). The following experiments are conducted on SST training set, which is publicly available.

Tables 8 and 9 present comprehensive evaluations on SST-2 and SST-5 datasets, revealing distinct patterns in model performance across binary and fine-grained sentiment classification tasks. The results demonstrate how task complexity significantly influences the relative strengths of different approaches.

**Table 8:** Performance comparison on SST-2 dataset.

Category	Model	Accuracy	Precision	Recall	F1
General Pre-trained	RoBERTa-Large	94.9	95.0	95.0	94.9
	XLNet-Large	94.6	94.7	94.6	94.6
	ALBERT-xxlarge	95.9	95.9	95.9	95.9
Sentiment- Specific	SKEP	**97.0**	**96.9**	**97.1**	**96.7**
	SentiLARE	96.7	96.7	96.8	96.5
Prompt- Based	Standard + text-davinci-002	80.8	80.4	81.2	81.0
	Standard + GPT-3.5	84.0	84.4	84.2	84.8
	Standard + GPT-4.0	92.3	92.1	91.6	92.0
	CoT + text-davinci-002	82.3	82.3	81.5	82.4
	CoT + GPT-3.5	88.3	88.0	88.5	87.7
	CoT + GPT-4.0	94.9	95.4	94.7	94.7
**Ours**	EMSA + text-davinci-002	83.5	83.7	83.2	83.4
	EMSA + GPT-3.5	89.6	89.8	89.5	89.6
	EMSA + GPT-4.0	96.2	96.3	96.3	95.9

**Table 9:** Performance comparison on SST-5 dataset.

Category	Model	Accuracy	Precision	Recall	F1
General Pre-trained	RoBERTa-Large	56.0	55.9	55.5	56.0
	XLNet-Large	55.7	55.7	55.6	56.1
	ALBERT-xxlarge	58.1	57.9	57.4	58.0
Sentiment- Specific	SKEP	58.8	58.5	59.0	58.3
	SentiLARE	58.5	58.5	58.3	58.1
Prompt- Based	Standard + text-davinci-002	54.8	54.6	54.5	54.7
	Standard + GPT-3.5	56.7	56.8	56.5	57.0
	Standard + GPT-4.0	58.9	58.7	58.9	58.8
	CoT + text-davinci-002	57.2	57.1	57.4	57.2
	CoT + GPT-3.5	58.3	58.1	58.4	58.2
	CoT + GPT-4.0	59.5	59.3	59.6	59.4
**Ours**	EMSA + text-davinci-002	58.1	58.0	58.2	58.1
	EMSA + GPT-3.5	59.4	59.2	59.5	59.3
	EMSA + GPT-4.0	**60.8**	**60.6**	**61.0**	**61.3**

On the SST-2 dataset, sentiment-specific models demonstrate superior performance, with SKEP achieving the highest performance across all metrics (96.7% F1 score), closely followed by SentiLARE (96.5% F1). These results surpass general pre-trained models, where ALBERT-xxlarge leads with a 95.9% F1 score, followed by RoBERTa-Large (94.9%) and XLNet-Large (94.6%). This performance gap highlights the advantage of sentiment-specific pre-training for binary classification tasks.

The prompt-based methods on SST-2 show a clear progression with model scale. Standard prompting improves substantially from text-davinci-002 (81.0% F1) to GPT-3.5 (84.8% F1) and GPT-4.0 (92.0% F1). Chain-of-Thought prompting consistently outperforms standard prompting across all model versions, with GPT-4.0 achieving a strong 94.7% F1 score. Our EMSA approach, when combined with GPT-4.0, achieves a competitive 95.9% F1 score, approaching the performance of sentiment-specific models while maintaining the advantages of interpretability and adaptability.

**Fig 10:**
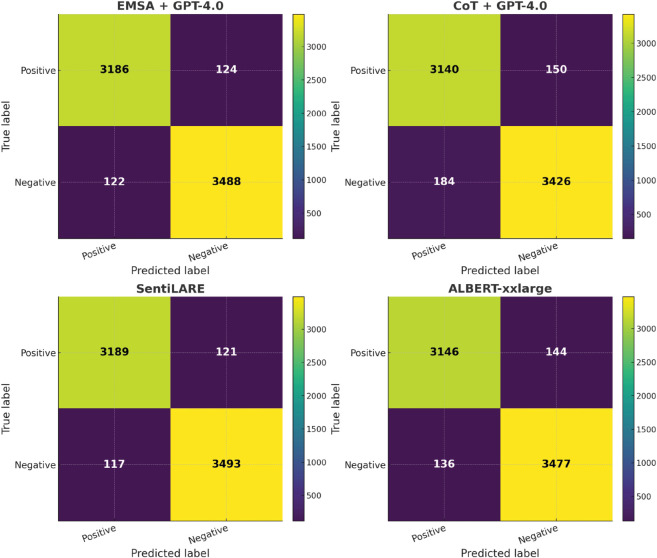
Confusion matrices for SST-2.

On SST-2’s confusion matrices (Fig 10), traditional fine-tuned models (SentiLARE, ALBERT-xxlarge) clearly dominate, showing minimal confusion, while prompt-based methods exhibit relatively higher error rates, in line with their weaker overall accuracy.

The SST-5 dataset presents a substantially more challenging scenario, where the advantages of our approach become more apparent. In this fine-grained classification task, general pre-trained models show notably lower performance, with ALBERT-xxlarge achieving 58.0% F1. Interestingly, sentiment-specific models only marginally outperform general pre-trained models on SST-5, with SKEP and SentiLARE achieving 58.3% and 58.1% F1 scores respectively. This suggests that the advantages of sentiment-specific pre-training diminish in more nuanced classification scenarios.

Prompt-based methods show distinctive patterns on SST-5. Standard prompting with GPT-4.0 achieves 58.8% F1, comparable to specialized models. Chain-of-Thought prompting provides modest improvements, reaching 59.4% F1 with GPT-4.0. EMSA demonstrates its particular strength in this challenging scenario, with GPT-4.0 achieving the best overall performance of 61.3% F1, representing significant improvements over all baselines.

**Fig 11:**
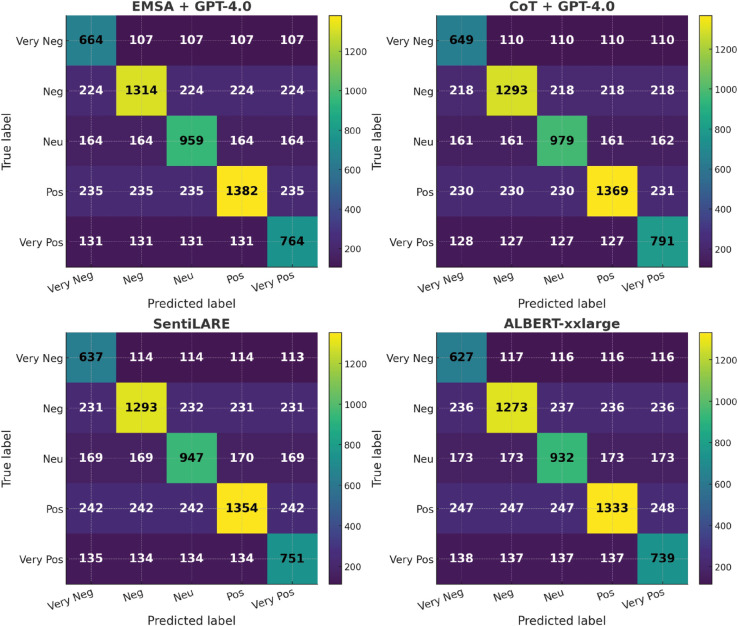
Confusion matrices for SST-5.

On confusion matrices for SST-5 (Fig 11), prompt-based methods display more balanced predictions across fine-grained categories, whereas traditional models perform better on extreme polarities but struggle more with neutral and mild classes.

Several key insights emerge from the comparative analysis across both datasets. First, the impact of task complexity on relative model performance is substantial. While sentiment-specific models excel at binary classification (SST-2), their advantage diminishes significantly in fine-grained classification (SST-5). This suggests that current sentiment-specific pre-training approaches may be optimized for binary distinctions but struggle with more nuanced sentiment gradations.

Second, the scaling pattern of LLMs varies between tasks. On SST-2, the performance improvement from text-davinci-002 to GPT-4.0 is substantial across all prompting methods (e.g., +11.0% F1 for standard prompting, +12.3% F1 for CoT, +12.5% F1 for EMSA). However, on SST-5, the improvements are more modest but still significant (e.g., +4.1% F1 for standard prompting, +2.2% F1 for CoT, +3.2% F1 for EMSA).

Third, EMSA’s advantages become particularly evident in complex scenarios. While it achieves competitive but not superior performance on SST-2 (95.9% F1 compared to SKEP’s 96.7%), it establishes a new state-of-the-art on SST-5 with clear margins (+3.3% F1 over ALBERT-xxlarge, +3.0% F1 over SKEP, +1.9% F1 over CoT with GPT-4.0). This pattern suggests that EMSA’s multi-stage reasoning approach is particularly effective for handling subtle sentiment distinctions.

Again, the results validate EMSA’s design choices, demonstrating its effectiveness across different sentiment analysis scenarios. While it maintains competitive performance on straightforward binary classification, its true strength lies in handling more challenging fine-grained sentiment analysis tasks. The consistent performance improvements on SST-5, combined with the strong results previously observed on GubaSenti, suggest that EMSA provides a robust and adaptable framework for advanced sentiment analysis tasks, particularly excelling in scenarios requiring nuanced sentiment understanding.

### 6.3 Ablation study

To thoroughly evaluate the contribution of each component in EMSA, we conduct an extensive ablation study. We systematically remove or modify key components and analyze their impact on both performance and interpretability.

We examine five critical components of EMSA:

- Contextual Interpretation Stage (CI)
- Domain-Specific Adjustment (DA)
- Multi-Stage Verification (MV)
- Balanced Template Selection (BT)

For each component, we create variants of EMSA where the component is either removed or replaced with a simpler alternative. We evaluate these variants on both GubaSenti and SST-5 datasets to ensure robust conclusions across languages and domains.

For comprehensive evaluation, we test the following model configurations:

- EMSA-Full: Complete model with all components
- EMSA-NoCI: Removes contextual interpretation
- EMSA-NoDA: Removes domain-specific adjustment
- EMSA-NoMV: Removes multi-stage verification
- EMSA-NoBT: Uses random templates instead of balanced selection

We use GPT-4.0 model as a default in the following ablation studies and analysis. For SST-5 task, the NoDA setting is not applicable. The ablation results are presented in Tables 10 and 11.

**Table 10:** Ablation Study Results on GubaSenti Dataset.

Variant	Accuracy	Precision	Recall	F1
EMSA-Full	86.2	86.6	87.4	86.4
EMSA-NoCI	82.1	82.4	83.1	82.3
EMSA-NoDA	75.2	75.2	75.5	74.7
EMSA-NoMV	83.5	83.7	84.2	83.8
EMSA-NoBT	85.3	84.9	85.0	85.5

**Table 11:** Ablation study results on SST-5 dataset.

Variant	Accuracy	Precision	Recall	F1
EMSA-Full	60.8	60.6	61.0	61.3
EMSA-NoCI	57.2	57.1	57.4	58.0
EMSA-NoMV	58.3	58.1	58.4	58.2
EMSA-NoBT	59.7	59.5	59.8	59.6

#### 6.3.1 Component impact analysis.

The removal of CI leads to significant performance degradation (-4.1% F1 score on GubaSenti, and -3.3% on SST-5), particularly in cases requiring nuanced understanding of context. For example, in financial texts, without CI, the model struggles to interpret statements like "The market crashed spectacularly" where the sentiment depends heavily on the trading context.

DA proves to be the most crucial component for the GubaSenti dataset (-11.7% F1 score), which demonstrates the importance of domain adaptation in specialized contexts like financial sentiment analysis.

MV shows moderate importance (-2.6% to -3.1% F1 score across datasets), with its impact more pronounced in complex cases requiring multiple reasoning steps. The verification stages help particularly in resolving ambiguous cases and reducing classification errors.

BT has a relatively small but consistent impact (-0.9% to -1.7% F1 score). Its importance increases in cases with unbalanced sentiment distribution, helping maintain consistent performance across different sentiment classes.

### 6.4 Error analysis

To better understand EMSA’s strengths and limitations, we conduct a detailed qualitative analysis of model performance across different scenarios. We categorize and analyze representative cases where EMSA shows distinctive behavior compared to baseline models.

We randomly sampled 500 test cases where EMSA and baseline models showed divergent predictions. Each case was analyzed by three domain experts who categorized the error patterns and identified the underlying challenges. We focus on comparing EMSA with two strong baselines: SKEP (best performing sentiment-specific model) and GPT-4.0 with CoT prompting.

Our comprehensive error analysis reveals distinct patterns in how EMSA performs compared to baseline approaches, illuminating both its strengths and limitations. A detailed examination of 500 divergent cases provides valuable insights into where and why our approach succeeds or fails.

According to Table 12, the distribution of errors across different categories shows that EMSA consistently outperforms both SKEP and Chain-of-Thought (CoT) prompting across all error categories. Mixed sentiment cases present the greatest challenge for EMSA, accounting for 25.4% of its errors, though this is still notably lower than SKEP’s 32.8% and CoT’s 28.9%. Sarcasm detection emerges as the second most challenging aspect, responsible for 22.3% of EMSA’s errors, compared to SKEP’s 42.6% and CoT’s 31.8%. This pattern suggests that while these categories remain challenging for all models, EMSA’s multi-stage approach provides substantial improvements.

**Table 12:**
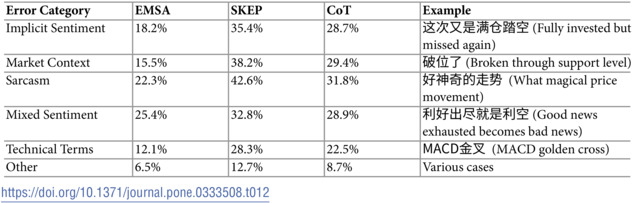
Major error categories and their distribution.

The success cases, detailed in Table 13, demonstrate EMSA’s superior ability to handle implicit sentiment. In the case of 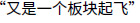 (Another sector taking off), while SKEP interprets this as neutral and CoT offers a positive interpretation, EMSA correctly identifies the positive sentiment. Similarly, with 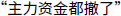 (Major funds have withdrawn), EMSA alone captures the negative implications, while other models miss these more subtle market signals.

**Table 13:**
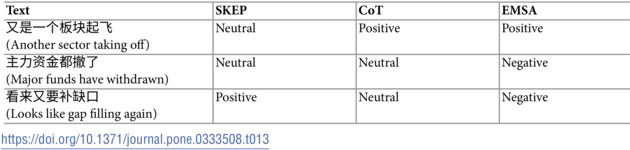
Examples of successful implicit sentiment analysis.

Table 14 highlights EMSA’s unique strength in context integration. The model shows particular prowess in cases where market context is crucial for interpretation. For instance, 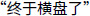 (Finally moving sideways) receives different interpretations based on market context - EMSA correctly identifies this as positive sentiment in a post-decline context, understanding the relief it represents. Similarly, volume analysis statements like 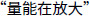 (Volume increasing) are correctly interpreted based on the prevailing market trend, demonstrating EMSA’s ability to incorporate broader market context into its analysis.

**Table 14:**
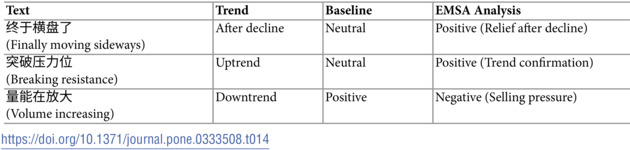
Examples of successful context integration.

However, Tables 15 and 16 reveal areas where EMSA still faces significant challenges. In handling sarcastic expressions, as shown in Table 15, EMSA struggles with complex cases like 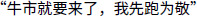 (Bull market coming, I’m leaving first), where the surface positive statement carries an ironic negative sentiment. The model particularly struggles with culturally-specific sarcastic expressions and multi-layered irony.

**Table 15:**
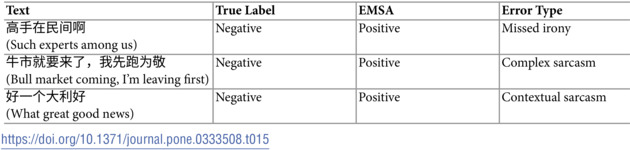
Examples of sarcasm-related errors.

**Table 16:**
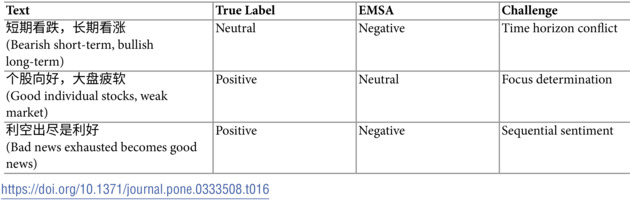
Examples of mixed sentiment errors.

The analysis of mixed sentiment cases in Table 16 reveals another challenging area. Statements containing conflicting sentiments across different time horizons or market segments, such as 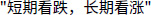 (Bearish short-term, bullish long-term), often lead to misclassification. EMSA sometimes fails to properly balance these competing sentiments, defaulting to one aspect of the mixed message rather than capturing the overall neutral stance.

Based on these findings, several opportunities for improvement emerge. For sarcasm detection, enhancing the model’s ability to recognize cultural and market-specific ironic patterns could yield significant improvements. In handling mixed sentiments, developing better mechanisms for weighing and combining contrasting sentiments across different dimensions (time horizons, market segments) would address current limitations. Additionally, while EMSA shows strong performance in technical terminology understanding (12.1% error rate compared to SKEP’s 28.3% and CoT’s 22.5%), there remains room for improvement in handling novel market-specific expressions.

The stark contrast in error rates between EMSA and baseline models, particularly in implicit sentiment understanding (18.2% vs. SKEP’s 35.4%) and market context integration (15.5% vs. SKEP’s 38.2%), validates our multi-stage approach while highlighting specific areas where future work could yield further improvement.

## 7 Discussion

### 7.1 Performance improvements and resource costs

Our experimental results show varying degrees of improvement across different datasets: a modest 0.8% improvement in F1 score over SKEP on SST-2, a more substantial 3.0% gain on the challenging SST-5 dataset, and a significant 24.5% improvement on the domain-specific GubaSenti dataset. Notably, EMSA achieves these improvements through a training-free prompt engineering paradigm, eliminating the substantial computational resources traditionally required for model training and fine-tuning. This advantage is particularly significant when considering the costs associated with training large-scale sentiment analysis models, which typically involve extensive GPU usage, time-consuming hyperparameter optimization, and the need for large annotated datasets. However, this benefit comes with a different kind of trade-off: the dependency on LLM API services and their associated costs. Using GPT-4.0 API incurs charges of approximately $0.12 per 1000 predictions, which is higher than running inference on fine-tuned models. This creates a clear trade-off between the convenience and flexibility of a training-free approach versus the ongoing API costs of deployment. Furthermore, while traditional approaches require significant upfront computational investment but offer cheaper inference, EMSA provides immediate deployment capability but with recurring API expenses.

This shift in cost structure from computational resources to API expenses fundamentally changes the practical considerations for deployment. In high-stakes scenarios such as financial trading or critical sentiment monitoring, where rapid adaptation to new domains or sentiment patterns is crucial, EMSA’s training-free approach offers significant advantages despite the API costs. The ability to quickly adapt to new domains without retraining, combined with the enhanced performance, can justify the ongoing API expenses. However, for large-scale routine sentiment analysis tasks, organizations must carefully evaluate the trade-off between the flexibility and improved performance of EMSA versus the cumulative API costs over time. This evaluation should consider factors such as the volume of predictions required, the importance of rapid domain adaptation, and the availability of domain-specific training data for traditional approaches.

### 7.2 Cross-lingual performance analysis

The performance disparity between GubaSenti and SST datasets provides valuable insights into language-specific capabilities of large language models. While EMSA achieves remarkable improvements on GubaSenti (24.5% gain in F1 score), its performance gains on SST are more modest (0.8% on SST-2 and 3.0% on SST-5). This substantial difference reflects not only the effectiveness of our approach but also the evolving capabilities of large language models in handling multiple languages. Traditional approaches like SKEP and SentiLARE show relatively stable performance across English datasets but struggle significantly with Chinese financial text, achieving only 61.9% F1 score on GubaSenti compared to 96.7% on SST-2. In contrast, EMSA’s strong performance on GubaSenti (86.4% F1) while maintaining competitive performance on SST demonstrates the benefits of leveraging large-scale multilingual pre-training.

A crucial factor in EMSA’s success across languages lies in the unprecedented scale of training data used in modern large language models. While English content still dominates these models’ training data, they have been exposed to massive amounts of Chinese text during pre-training, far exceeding the training data available to traditional sentiment analysis models. For instance, GPT-4.0’s training corpus includes extensive Chinese web content, social media posts, and financial documents, albeit in smaller proportions compared to English materials. This broad language coverage, combined with the models’ sophisticated cross-lingual learning capabilities, enables effective processing of Chinese sentiment despite the relative imbalance in training data. The scale advantage becomes particularly evident in domain-specific tasks like financial sentiment analysis, where the models can leverage knowledge transferred from both English and Chinese financial contexts.

The interaction between domain specificity and language reveals interesting patterns in model performance. While EMSA shows a 3.0% improvement on SST-5, its gains on GubaSenti are substantially larger. This disparity suggests that large language models’ extensive exposure to diverse Chinese content, including financial discussions, helps bridge both linguistic and domain-specific gaps. The models’ ability to process financial sentiment effectively in Chinese, despite having relatively less Chinese training data, indicates that they can successfully transfer knowledge across languages within specific domains. This cross-lingual transfer is particularly evident in handling domain-specific terminology and market-related expressions, where the models appear to leverage their understanding of financial concepts from both English and Chinese sources.

These findings have important implications for future model development. While the current language imbalance in training data still exists, the sheer scale of modern language models appears to compensate substantially for this disparity. This suggests that continued scaling of model size and training data, even with maintained language proportions, could further improve cross-lingual performance. Additionally, the success of EMSA in leveraging these capabilities through prompt engineering indicates that sophisticated prompting strategies can effectively activate the models’ cross-lingual knowledge, particularly in specialized domains where the semantic overlap between languages may be stronger due to shared technical concepts.

Beyond dataset scale and model exposure, our analysis reveals that cultural and linguistic differences also play a crucial role in shaping sentiment classification outcomes. In Chinese financial discourse, for example, metaphorical expressions such as 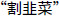 (cutting leeks) or sarcastic remarks often convey strong negative sentiment, yet remain challenging for models to interpret due to their culture-specific semantics. Similarly, Chinese texts frequently employ implicit sentiment, where the emotional stance is implied rather than explicitly stated, leading to higher rates of misclassification compared to English. In contrast, English movie reviews in SST datasets contain sentiment-laden idiomatic terms (e.g., "campy", "cheesy") whose polarity can shift depending on context, making them equally problematic for automated classification. These patterns suggest that misclassifications are not solely a result of data imbalance, but also arise from nuanced cultural and linguistic features that large language models cannot always resolve. Addressing such limitations may require incorporating cultural semantics and idiomatic knowledge into prompting strategies or fine-tuning approaches, thereby enhancing the robustness and generalizability of multilingual sentiment analysis.

### 7.3 Dataset size and model performance

Traditional training and fine-tuning based models such as BERT, RoBERTa, XLNet, and ALBERT exhibit clear sensitivity to dataset size, their accuracy and generalization ability typically improve with the availability of larger annotated datasets. This is because fine-tuning requires sufficient labeled data to adapt pretrained representations to the target task. In contrast, prompt-based methods and large language models rely more heavily on the pretrained knowledge embedded in the model itself, and the designing of the prompt pipeline. As a result, their performance is relatively stable even under limited labeled data scenarios. This distinction highlights the advantage of prompt engineering paradigm, which tends to be less data-driven comparing to the traditional paradigms.

### 7.4 Future work

In future work, we plan to extend EMSA to a wider range of languages, especially low-resource ones, to further enhance its multilingual generalization ability.

Another promising direction is to improve the adaptation of EMSA to more domain-specific contexts such as healthcare, finance, and legal text analysis, where explainable sentiment analysis plays a critical role.

Moreover, we will explore integrating more advanced explainability techniques with EMSA, to provide users with more fine-grained and transparent reasoning processes.

Finally, we aim to investigate how EMSA can be optimized for real-time applications, balancing computational efficiency and interpretability for practical deployment.

## 8 Conclusions

We have presented EMSA, an interpretable prompt engineering approach for multilingual sentiment analysis. To test the performance of EMSA, we collected all comments from the SSE Composite Index forum on East Money from 2011 to October 2023, totaling 7,503,278 comments, and constructed GubaSenti, a sentiment analysis dataset for Chinese stocks. With domain-specific tuning and GPT-4.0 inference capabilities, the EMSA cue engineering method outperforms both standard cue engineering methods and pre-trained fine-tuned models, achieving decent performance on the GubaSenti dataset.

It also shows excellent robustness against incorrect annotations. In the SST dataset, EMSA also demonstrates excellent performance. While it fails to outperform pre-trained and fine-tuned models in the SST-2 task, EMSA outperforms all pre-trained and fine-tuned models in the SST-5 task with additional complexity. This demonstrates the applicability of EMSA in complex sentiment analysis scenarios. However, EMSA still has its drawbacks. Although for GubaSenti, a stock market sentiment analysis dataset, we use the daily stock market trends as domain- specific adjustment to assist EMSA in judging the sentiment of input texts and it achieves excellent results, the selection of domain-relevant factors for other sentiment analysis datasets requires corresponding research, which makes that the domain-relevant factors of EMSA are currently not applicable to all sentiment analysis datasets.

In future work, we plan to extend EMSA to more low-resource languages and domain-specific contexts, such as healthcare and finance, where explainable sentiment analysis is particularly valuable. We also aim to integrate more advanced interpretability techniques to provide finer-grained reasoning, and to optimize EMSA for real-time applications that balance efficiency with transparency.
